# Increasing Plant-Based Meat Alternatives and Decreasing Red and Processed Meat in the Diet Differentially Affect the Diet Quality and Nutrient Intakes of Canadians

**DOI:** 10.3390/nu12072034

**Published:** 2020-07-09

**Authors:** Hassan Vatanparast, Naorin Islam, Mojtaba Shafiee, D. Dan Ramdath

**Affiliations:** 1 College of Pharmacy and Nutrition, University of Saskatchewan, Saskatoon, SK S7N 4Z2, Canada; naorin.islam@usask.ca (N.I.); mos866@usask.ca (M.S.); 2 School of Public Health, University of Saskatchewan, Saskatoon, SK S7N 4Z2, Canada; 3 Guelph Research and Development Centre, Agriculture and Agri-Food Canada, Guelph, ON N1G 5C9, Canada; dan.ramdath@canada.ca

**Keywords:** red and processed meat, plant-based meat alternatives, protein intake, diet modeling, Canada’s food guide, planetary health diet

## Abstract

Current evidence suggests a link between red and processed meat consumption and the risk of various cancers and other health outcomes. Using national survey data from the Canadian Community Health Survey (CCHS)-Nutrition 2015, we aimed to model a dietary scenario to assess the potential effects of increasing the intake of currently consumed plant-based meat alternatives by 100% and decreasing the consumption of red and processed meat by 50% on the diet quality and nutrient intakes of Canadians (≥1 year). This dietary scenario had no significant impact on dietary energy intake (*p* > 0.05), but resulted in a significant increase in the dietary intakes of fibre, polyunsaturated fatty acids, magnesium, and dietary folate equivalents (*p* < 0.05). On the other hand, this dietary scenario was accompanied by a significant decrease in protein (from 77.8 ± 0.6 g to 73.4 ± 0.6 g), cholesterol, zinc, and vitamin B12 intake (*p* < 0.05). Further, based on Nutrient Rich Food (NRF) scores, the overall nutritional value of the simulated diet was higher than the baseline diet. Our modeling showed that the partial replacement of red and processed meat with plant-based alternatives improves overall diet quality but may adversely affect the intake of some micronutrients, especially zinc and vitamin B12.

## 1. Background

As a macronutrient, protein is an indispensable component of the human diet and serves important physiological roles, including immune and endocrine function, metabolism, and muscle and bone growth [1]. According to the most recent Dietary Guidelines for Americans (2015–2020), the Recommended Dietary Allowance (RDA) for protein intake in adults is 0.8 g/kg of body weight, which is 56 g/day for a 70 kg individual or about 10% of energy intake [2]. Dietary protein may be derived from animal sources, plant sources, or a combination of both. Approximately two-thirds of protein intake in Western countries originates from animal sources, and the remaining one-third comes from plant sources [3]. Meat (i.e., red meat, poultry, game, processed meat, and offal) is the major source of animal protein intake (38%), and grains are the most important contributing food group for plant protein intake (17%) in Western countries [3]. Although animal sources of protein are of higher quality, a growing body of evidence suggests that diets high in red and processed meat are associated with negative environmental impacts and poorer health outcomes [4,5,6,7,8]. In a population-based cohort study with 16 years of follow-up, Etemadi and colleagues found an association between high intakes of red and processed meat and elevated all-cause mortality and mortality from most major causes: cancer, diabetes, cardiovascular disease, kidney disease, liver disease, and respiratory diseases [4]. In a prospective cohort study with 32 years of follow-up, protein intakes from animal and plant sources were assessed in relation to all-cause and cause-specific mortality among over 131,000 men and women. The authors reported that replacing protein from animal sources, especially processed red meat, with plant-based protein sources was associated with substantially reduced overall mortality [9]. On the basis of these observations and the positive health effects of the regular intake of plant-based foods on human health, the new Canada’s Food Guide (2019) has merged the former “meat and alternatives” and “milk and alternatives” food groups into one group, known as “protein foods.” This version of Canada’s Food Guide has also placed a major emphasis on plant-based sources of protein [10]. The recently released EAT-Lancet Commission report (2019) also showed that the transformation to healthy diets by 2050 will require substantial dietary shifts. This includes more than doubling the consumption of healthy foods such as legumes, nuts, fruits, and vegetables, and a greater than 50% reduction in the consumption of less healthy foods such as red meat and added sugars [11]. According to the EAT-Lancet report, a diet rich in plant-based foods and with fewer animal source foods not only improves human health, but also confers environmental benefits [11]. In this regard, Westhoek et al. reported that replacing 25–50% of animal-derived foods with plant-based foods on a dietary energy basis in the European Union would result in a 40% reduction in the intake of saturated fat, a 25–40% reduction in greenhouse gas emissions, a 40% reduction in nitrogen emissions, and 23% per capita less use of cropland for food production [12]. This is the first study to examine the impact of doubling the consumption of plant-based meat alternatives and reducing the consumption of red and processed meat by 50% on the nutrient intake of Canadians.

The primary objective of the present study was to use national survey data to model a dietary scenario to assess the potential effects of increasing the intake of plant-based meat alternatives and decreasing the consumption of red and processed meat on the diet quality and nutrient intakes of Canadians. To accomplish this goal, we conducted a diet modeling exercise to increase the amount of currently consumed plant-based meat alternatives (i.e., legumes, seeds, and nuts) by 100% while decreasing red and processed meat consumption by half. We also aimed to (1) determine the percent contribution of red and processed meat to daily energy and nutrient intake among red and processed meat consumers, and (2) determine the percent contribution of plant-based meat alternatives to daily energy and nutrient intake among plant-based meat alternatives consumers.

## 2. Subject and Method

### 2.1. Study Population and Dietary Data Collection

In this study, we used nationally representative data from the Canadian Community Health Survey–Nutrition 2015. This cross-sectional survey data was derived from Canadians 1 year and over across ten provinces. Individuals living in aboriginal settlements and reserves, Canadian forces employees who work full time, and the institutionalized population were not included in this survey [13]. The data were collected from 2 January 2015 to 31 December 2015 from the respondents directly, with a response rate of 62%. Dietary data such as frequency, time, location, amount of consumption, food items consumed, and supplement consumption were collected from the survey respondents. The dietary data were collected using two 24 h recalls. The first 24 h recall was collected from 20,487 individuals, and the second recall was collected from a subsample of the first recall. We used the dietary intake derived from the first recall in this study. A computer-assisted personal interview (CAPI) was used to interview the respondents at first recall, and a computer-assisted telephone interview (CATI) was used to collect the second dietary recall. Children 1–5 years in age participated in the interview using a proxy (i.e., the information was provided by the children’s guardian or parents). Children 6–11 years responded to the survey with the help of their parents, and individuals 12 years and above were interviewed on their own. The proxy interview was also used for individuals 6 years and above who were not able to provide information by themselves due to physical or mental health. The detailed information on this survey methodology can be found at the Statistics Canada website [14].

### 2.2. Analytical Sample

This study included 34,005,286 individuals (weighted frequency) who were 1 year or older. Red and processed meat consumers were defined as individuals who reported any beef, game, and organ meats; other meats such as pork, veal, and lamb; and processed meat consumption, based on the 2007 Canada Food Group (CFG) food classification. Plant-based meat alternatives consumers were defined as participants who reported consuming any legumes, nuts, and seeds based on the CFG classification [15]. The details of these food groups were mentioned in the appendix. The sociodemographic differences between red and processed meat and plant-based meat alternatives consumers and non-consumers were reported by two age groups: 1–18 years (children and adolescents) and ≥18 years (adults).

### 2.3. Sociodemographic Characteristics

Sociodemographic variables such as age, sex, ethnicity, food security, education status, marital status, body mass index (BMI), obesity, immigration status, and urban/rural residence were reported. The variables were categorized as sex (female, male), ethnicity (white, non-white), education (university degree, non-university degree), marital status (married, not married), food secured (yes, no), overweight or obesity (yes, no), immigrant (yes, no), and residence (urban/rural).

### 2.4. Total Daily Energy and Nutrient Intake and Simulated Intake

The daily nutrient and energy intake were reported for Canadians ≥1 year who were red and processed meat or plant-based meat alternatives consumers. We also reported the simulated daily nutrient and energy intake as well as the Nutrient Rich Food (NRF) score and compared the results with the baseline diet. The simulated intakes were derived by doubling the daily intake of legumes, nuts, seeds, and reducing the daily intake of beef, game, and organ meats; other meats such as pork, veal, and lamb; and processed meat by half.

### 2.5. Nutrient Density

To evaluate the diet quality of Canadians, we used the NRF Index 9.3, which measures the nutritional quality per serving [16]. The NRF index is based on “nine nutrients to encourage” (fibre, protein, calcium, vitamin D, iron, magnesium, potassium, vitamin A, vitamin C) and “three nutrients to limit” (saturated fatty acid, total sugar, and sodium). The index was calculated by subtracting the sum of the percentage of maximum recommended values for “three nutrients to limit” from the percentage daily values for the “nine nutrients to encourage” [17]. The maximum possible NRF score is 900, which reflects the best diet quality. If the percentages daily values of “nutrients to encourage” exceed 100, the values were truncated at 100, and if the percentages of the daily values of “nutrients to limit” were less than the daily value, they were reported as zero. The nutrients daily values of the Canadians can be found at the Government of Canada website (Table 1) [18]. Since Canada does not have a daily value of protein, we used a 10% value of energy [19]. The daily values used to estimate the NRF 9.3 are as follows:

### 2.6. Statistical Analyses

The statistical analyses presented in this study were performed using SAS (SAS Institute, version 9.4, Cary, NC, USA). All the estimates were weighted and bootstrapped based on Statistics Canada’s guideline for survey analysis to obtain estimates at the population level [20]. We represented the results as mean ± SE for continuous variables and as % ± SE for categorical variables. The sociodemographic differences between red and processed meat consumers and non-consumers and plant-based meat alternatives consumers and non-consumers were tested using the chi-square test for categorical variables (sex, ethnicity, education status, marital status, food security, overweight/obesity, residence, immigrant status) and by F-statistics for continuous variables (age, BMI, BMI *z*-score). We calculated the mean percent nutrient contribution from red meat and plant-based meat alternatives to daily nutrient intake among red meat consumers and plant-based meat alternatives consumers separately. The differences in daily nutrient intake between the Canadian Community Health Survey (CCHS) 2015 and the simulated intake were tested using a 95% confidence interval non-overlap technique [21]. We set the alpha at 0.05 for all the statistical analyses.

## 3. Results

### 3.1. Prevalence of Red Meat and Plant-Based Meat Alternatives Consumption

Overall, 41.7% of Canadians reported consuming red and processed meat and 14.5% reported consuming plant-based meat alternatives (Table 2). The sociodemographic differences between red and processed meat consumers and non-consumers and plant-based meat alternatives consumers and non-consumers are reported in Table 2. Among children, red and processed meat consumers were more likely than non-consumers to come from a household without a university degree (*p* < 0.05). About 40% of red and processed meat consumers and 48% of non-consumers came from a household with a university graduate (*p* < 0.05). The red and processed meat consumers were significantly (*p* < 0.05) older compared to non-consumers (9.3 ± 0.1 vs. 8.8 ± 0.1 years, respectively). Among adults, the percentage of males was significantly (*p* < 0.05) higher in the red and processed meat consumers compared to the non-consumers (56.6 ± 1.0 vs. 45.7 ± 0.7, respectively). The red and processed meat consumers were less likely (*p* < 0.05) to come from a household with a university degree (33.4 ± 1.4 vs. 42 ± 1.2, respectively). Adult red and processed meat consumers were more likely (*p* < 0.05) to be food insecure compared to non-consumers (87.3 ± 0.8 vs. 89.4 ± 0.7, respectively). Moreover, adult consumers were more likely (*p* < 0.05) to be rural residents compared to non-consumers (80.5 ± 1.0 vs. 84 ± 1.0, respectively).

Among children and adolescents, there were significantly (*p* < 0.05) less males among the consumers of plant-based meat alternatives compared to the non-consumers (43.9 ± 3.0 vs. 50.9 ± 0.9, respectively). Further, there was a significantly (*p* < 0.05) higher percentage of university graduate households among the consumers compared with non-consumers (58.6 ± 3.1 vs. 42.9 ± 1.3, respectively). Among Canadian adults, the percentage of males was significantly (*p* < 0.05) lower among the consumers of plant-based meat alternatives compared to non-consumers (37.5 ± 1.9 vs. 52.3 ± 0.4, respectively). The plant-based meat alternatives consumers were more likely (*p* < 0.05) than the non-consumers to come from a household with a university degree (50.1 ± 2.3 vs. 36.5 ± 1.0, respectively). The percentage of non-white individuals was significantly (*p* < 0.05) higher among the consumers (75.9 ± 1.0) compared to the non-consumers (68.3 ± 2.2). The plant-based meat alternatives consumers were more likely (*p* < 0.05) than non-consumers to be married (68.3 ± 2.0 vs. 62.3 ± 0.9, respectively). The prevalence of food security was significantly (*p* < 0.05) higher among the plant-based meat alternatives consumers than the non-consumers (91.8 ± 1.1 vs. 87.9 ± 0.6, respectively). The percentage of immigrants among the consumers compared to non-consumers was significantly (*p* < 0.05) higher (34.6 ± 2.2 vs. 26.0 ± 1.0, respectively).

### 3.2. Daily Nutrient Intake and Percent Nutrient Contribution from Red and Processed Meat and Plant-Based Meat Alternatives

Table 3 presents the percent energy and nutrient contribution from red and processed meat and plant-based meat alternatives among the consumers of protein sources. In 2015, red and processed meat accounted for 12.0% of the daily energy intake among red meat consumers, and plant-based meat alternatives accounted for 12.7% of the daily energy intake among plant-based alternatives consumers. Red and processed meat contributed substantially to the daily intake of vitamin B12 (38.0%), zinc (33.1%), cholesterol (32.6%), protein (29.0%), and monounsaturated fatty acids (MUFA) (22.4%) among red and processed meat consumers. The top five nutrients provided by the plant-based meat alternatives among consumers were polyunsaturated fatty acids (PUFA, 27.0%), monounsaturated fatty acids (MUFA, 24.8%), total fat (21.4%), magnesium (20.3%), and dietary fibre (20.1%). Red and processed meat was a major contributor to saturated fat intake (21.2%) among the red and processed meat consumers, while plant-based meat alternatives accounted for 13.0% of daily saturated fat intake among the plant-based alternatives consumers. In addition, plant-based meat alternatives contributed to only 14.9% of protein intake among plant-based alternatives consumers, about half the amount provided by red and processed meat (29%).

### 3.3. Comparison of Daily Energy and Nutrient Intakes between CCHS 2015 and Simulated Results

Table 4 reports the comparison of daily energy and nutrient intakes between the CCHS 2015 and simulated results. There was no significant difference in energy intake between the results from CCHS 2015 (1863.4 ± 12.0 kcal) and the simulated results (1867.1 ± 12.6 kcal). Doubling the consumption of plant-based meat alternatives and reducing the intake of red and processed meat by half resulted in a significant decrease in protein (from 77.8 ± 0.6 g to 73.4 ± 0.6 g), cholesterol (from 261.8 ± 3.8 mg to 239.0 ± 3.8 mg), zinc (from 10.3 ± 0.1 mg to 9.4 ± 0.1 mg), and vitamin B12 (from 4.05 ± 0.06 mcg to 3.46 ± 0.05 mcg) intake. On the other hand, the simulation results showed a significant increase in dietary intakes of fibre (from 16.8 ± 0.2 g to 18.1 ± 0.2 g), PUFA (from 14.3 ± 0.2 g to 15.6 ± 0.2 g), magnesium (from 297.6 ± 2.1 mg to 315.4 ± 2.7 mg), and dietary folate equivalents (DFE) (from 437.5 ± 3.5 mcg to 454.8 ± 3.8 mcg). Diet quality, as reflected by NRF, also significantly increased in the simulated results (from 517.4 to 525.8, *p* < 0.05).

## 4. Discussion

In the present study, we aimed to model a dietary scenario to assess the potential impact of increasing the currently consumed plant-based meat alternatives (by 100%) and decreasing the currently consumed red and processed meat (by 50%) on the diet quality and nutrient intakes of Canadians. This dietary scenario resulted in a significant increase in the dietary intakes of fibre, PUFA, magnesium, and DFE, and was accompanied by a significant decrease in protein, cholesterol, zinc, and vitamin B12 intake. Further, based on the NRF scores, the overall nutritional value of the simulated diet was higher than the baseline diet.

Animal-based protein sources such as red meat generally contribute more protein than plant-based protein foods do [22]. Although nuts and certain types of beans (black and great northern beans) are comparable in their protein content to meat, vegetable products, certain types of beans (lima beans, pinto beans, and kidney beans), and soy-based tofu products have much lower protein contents [22]. Our results revealed that doubling the intake of plant-based meat alternatives and reducing the intake of red and processed meat by half resulted in a significant decrease in protein intake (−4.4 g/day). Using biophysical models and methods, Westhoek et al. examined the consequences of replacing 50% of animal-derived foods with plant-based foods on a dietary energy basis in the European Union [12]. In this regard, the authors developed three alternative diets by a 50% reduction in the consumption of beef, pig meat, poultry, dairy, and eggs, which was compensated by an increase in cereals. Their results showed that the protein intake in the alternative diets was up to about 10% lower than the protein intake in the reference diet. However, even with a 50% reduction in all animal products, the mean protein intake in the European Union was still 50% higher than the dietary protein requirements [12]. Using baseline data from the PREMIER clinical trial (1999–2002), Lin et al. reported that animal-based proteins and plant-sourced protein foods comprised approximately 66% and 34% of the daily protein intake, respectively, among U.S. adults. However, age, sex, body weight status, and race significantly influenced the contribution patterns from different food groups [23]. Using dietary intake data from the National Health and Nutrition Examination Survey (NHANES) 2007–2010, it was found that poultry and meats, respectively, accounted for 10% and 9.5% of daily protein intake, while plant-based foods contributed to only 3.2% of daily protein intake in American men and women aged >19 years [24]. Cifelli et al. used national survey data from the NHANES 2007–2010 to assess the effects of increasing dairy foods or plant-based foods on the macronutrient intake and nutrient adequacy of U.S. children (2–18 years) and adults (≥19 years) [25]. The authors compared the usual diet at baseline with three dietary scenarios that increased intakes by 100%: (1) plant-based foods, (2) protein-rich plant foods (i.e., soy, nuts, seeds, legumes), and (3) dairy (i.e., milk, cheese, yogurt). In the first two scenarios, there was a commensurate decrease in animal products intake. Increasing the intake of plant foods by 100% resulted in a significant decrease in protein intake in both children (from 68.7 to 58.0 g/day) and adults (from 82.1 to 64.9 g/day). In contrast, doubling protein-rich plant-based foods did not impact any of the nutrients because they were consumed in very low quantities in the baseline diet [25]. Using the same national survey data, it was found that doubling the intake of plant-based foods (as currently consumed) and commensurate reductions in animal-based protein intake resulted in an about 22% decrease in protein intake per ideal body weight in males and females aged ≥51 years [26]. Although plant-based meat alternatives provide a lower quantity of protein than animal products do, the amounts of protein in plant-based (lacto-ovo-vegetarian diets with half of all protein from plant sources) or vegan food patterns are above the RDA [27]. Therefore, replacing animal-based proteins with plant-based meat alternatives would not result in a protein deficit.

Plant-based, protein-rich foods like legumes, nuts, and seeds are low in saturated fat content, free of trans fat, and cholesterol-free [28]. Although, compared to meat, nuts usually have an intermediate amount of saturated fat content (3.8–7.8 g/100 g serving), soy-based tofu products (0.5–0.8 g/100 g serving), beans (0.1–0.7/100 g serving), and vegetable products (less than 0.1/100 g serving) are very low in saturated fat content [22]. Our results showed that plant-based meat alternatives accounted for only 13% of saturated fat intake in plant-based alternatives consumers, while red and processed meat contributed to 21.2% of saturated fat intake in red and processed meat consumers. Further, when currently consumed plant-based meat alternatives were increased by 100% and red and processed meat were decreased by half, there was a significant decrease in the cholesterol intake (−22.8 mg/day) and a non-significant decrease in the saturated fat intake (−0.7 g/day). Westhoek et al. reported that replacing 50% of animal-derived foods (i.e., beef, pig meat, poultry, eggs, and dairy) with plant-based foods (i.e., cereals) on a dietary energy basis would result in a 40% reduction in the intake of saturated fat in the European Union, where the per capita intake of animal foods is high [12]. Using data from children, adolescents, and adults participating in the What We Eat in America (WWEIA) 2003–2004 and NHANES 2005–2006, Huth et al. found that cheese (16.5%), beef (8.5%), and milk (8.3%) are the three top sources of saturated fatty acids in the U.S. diet [29]. Using national survey data from the NHANES 2007–2010, it was found that increasing the amount of currently consumed plant-based foods by 100% and decreasing animal products on a gram per gram basis resulted in a significant decrease in saturated fat intake in both U.S. children (from 25.2 g/day at baseline to 20.1 g/day) and adults (from 26.2 g/day at baseline to 20.0 g/day) [25]. However, increasing protein-rich plant-based foods by 100% and a commensurate decrease in animal products had only a negligible effect on the saturated fat intake in both children and adults [25]. The same results were obtained in another study conducted in female adolescents aged 9–18 years, in which a similar approach was used [30]. During a 6-month randomized controlled trial, overweight and obese adults on a vegan diet had a significantly lower percentage of energy from saturated fat (2 and 6 months) and a lower intake of cholesterol (2 and 6 months) when compared with those on a vegetarian, semivegetarian, pescovegetarian, or omnivorous diet [31]. Since the majority of Canadians consume more than the recommended 10% of total energy intake from saturated fat [32], replacing animal-based proteins, especially red and processed meat, with plant-based meat alternatives could be an effective strategy in reducing saturated fat intake.

Unlike plant-based meat alternatives, which are rich sources of carbohydrates such as fibre, starch, and oligosaccharides, meat is very low in carbohydrates and contains no dietary fibre [22,33]. Our results revealed that increasing the currently consumed plant-based meat alternatives by 100% and decreasing red and processed meat by half resulted in a significant increase in dietary fibre intake (+1.3 g/day) among the Canadian population. Cifelli et al. showed that doubling the currently consumed plant-based foods in the American diet significantly raised dietary fibre intake in children (from 13.5 g/day at baseline to 18.2 g/day) and adults (from 16.4 g/day at baseline to 21.6 g/day) [25]. In this dietary scenario, the percentage of children and adults with dietary fibre intakes above the adequate intake (AI) increased 5.1 and 2.6 times, respectively, compared to the baseline. However, no significant difference was observed in the dietary fibre intake between the usual diet and the dietary scenario that increased protein-rich plant foods by 100% [25]. Using the same national survey data, Demmer et al. also reported that doubling the amount of currently consumed plant-based foods increased the intake of dietary fibre by about 4 g/day among adolescent females [30]. However, the impact of doubling protein-rich plant foods on dietary fibre intake was negligible, since they were consumed in very low quantities at the baseline [30]. Moreover, in a 6-month randomized controlled trial, overweight and obese adults on a vegan diet had significantly greater intakes of dietary fibre (31.0 g/day), compared with participants on a vegetarian (26.7 g/day), pescovegetarian (21.0 g/day), semivegetarian (17.6 g/day), or omnivorous diet (17.6 g/day) at 2 months [31]. Furthermore, most Canadian men and women consume only about half of the recommended daily intake of dietary fibre (25 g/day for women and 38 g/day for men) [34]. Therefore, increasing the intake of plant-based meat alternatives could assist in increasing dietary fibre intake in the Canadian population.

Meat and meat products are valuable sources of some micronutrients, especially zinc, iron, and vitamin B12, which are known to be nutrients of concern in the diet of vegetarians [35,36]. In our dietary scenario, the partial replacement of red and processed meat with plant-based meat alternatives resulted in a significant decrease in the dietary intake of zinc (−0.9 mg/day) and vitamin B12 (−0.59 mcg/day), but had no impact on iron intake. In a study conducted in representative population samples of U.S. adults of five ethnic/racial groups, the contribution of meat was found to be substantial for vitamin B12 (19.7–40%) and zinc (11.1–29.3%) and, to a lesser extent, for iron (4.3–14.2%) [37]. In a cross-sectional study of female university students, Fayet et al. found that the avoidance of animal foods adversely affected the intakes of zinc, bioavailable iron, selenium, vitamin B12, and omega-3 fatty acids [38]. Doubling the intakes of plant-based foods with commensurate decreases in animal products was accompanied by increases in iron and folate intakes and decreases in zinc, calcium, and vitamin D intakes among female adolescents [30]. The same dietary scenario resulted in significant decreases in calcium and vitamin D intakes and significant increases in iron, magnesium, and folate intakes among U.S. children and adults. However, similar to our findings, increasing protein-rich plant foods by 100% had no significant impact on the dietary intake of iron [25]. In a 6-month randomized controlled trial, overweight and obese adults on an omnivorous diet had a significantly higher zinc intake than the participants on a vegan, vegetarian, pescovegetarian, or semivegetarian diet at both 2 and 6 months. There was no significant difference in iron intake at any time point between the adults following different dietary interventions [31]. It should be noted that, although plant-based meat alternatives, especially beans and nuts, are high in iron content [22], the form of this iron is non-heme, which is less bioavailable than heme iron [39]. Further, increasing the intake of phytate-containing legumes may compromise the absorption of both non-heme iron and zinc [40]. Therefore, when replacing red and processed meat with plant-based meat alternatives, caution should be taken to avoid potential zinc, vitamin B12, and iron deficiencies.

### Strengths and Limitations

This study used nutrition data from a nationally representative survey of the Canadian population, CCHS-Nutrition 2015. A major strength of this study was the opportunity to investigate the nutritional impact of dietary change based on the reduction in the consumption of red and processed meat (by 50%) and increase in consumption of plant-based meat alternatives (by 100%), shortly after the introduction of Planetary Health Diet and new Canada’s Food Guide. We also acknowledge some limitations. First, the data regarding protein intake were obtained using a 24 h dietary recall, which is a self-report method subject to misreporting, especially in overweight and obese individuals (i.e., overestimating or underestimating dietary intake). Second, the comparisons in daily nutrient intake between CCHS 2015 and simulated intake were made by a 95% confidence interval overlapping method. Third, since we combined age and sex groups in this study, it is possible that certain results may vary for different age/sex groups.

## 5. Conclusions

In conclusion, the results of this modeling study revealed that the partial replacement of red and processed meat with plant-based meat alternatives could lead to the improvement in dietary intake of some nutrients such as dietary fibre, magnesium, folate, and PUFA. Such a dietary scenario could limit the intake of cholesterol among the Canadian population but would have only minor effects on the saturated fat and sodium intake. Moreover, this dietary scenario would enhance the overall nutritional value of the diet, as reflected by higher NRF scores. On the other hand, doubling the intake of plant-based meat alternatives and decreasing red and processed meat by half would result in a significant decrease in the dietary intake of protein, zinc, and vitamin B12. According to the literature, Western dietary patterns contain an excess of protein, and subtracting meat from current Western diets or replacing meat with plant-based alternatives would not result in a protein deficit [41]. When replacing red and processed meat with plant-based meat alternatives, caution should be taken to decrease the risk of deficiencies for specific micronutrients, especially zinc and vitamin B12. Furthermore, this study adds to the literature on the sociodemographic differences of red and processed meat and plant-based alternatives consumers and non-consumers, which could be used by policy makers to develop targeted strategies to increase the intake of plant-based sources of protein.

## Figures and Tables

**Table 1:** The daily values of nutrients in Canada [18].

Nutrients	Daily Value
Protein	50 g
Fibre	28 g
Calcium	1300 mg
Vitamin D	20 mcg
Vitamin A	900 mcg
Vitamin C	90 mg
Iron	18 mg
Magnesium	420 mg
Potassium	4700 mg
Total Sugar	100 g
Saturated Fatty Acid	20 g
Sodium	2300 mg

**Table 2:** Sociodemographic differences between the red meat and plant-based alternatives consumers and non-consumers among Canadian children and adults.

Characteristic	Red and Processed Meat (41.7%)				Plant-Based Meat Alternative (14.5%)			
	Children/Teens (1–18 y) *n* = 6,439,045		Adults (≥19 y) *n* = 11,013,278		Children/Teens (1–18 y) *n* = 6,439,045		Adults (≥19 y) *n* = 27,566,241	
	Consumer (Mean or %, SE)	Non-Consumer (Mean or %, SE)	Consumer (Mean or %, SE)	Non-Consumer (Mean or %, SE)	Consumer (Mean or %, SE)	Non-Consumer (Mean or %, SE)	Consumer (Mean or %, SE)	Non-Consumer (Mean or %, SE)
	*n* = 3,161,313	*n* = 3,277,732	*n* = 11,013,278	*n* = 16,552,963	*n* = 691,078	*n* = 5,747,967	*n* = 4,235,199	*n* = 23,331,042
Mean age ± SD (y)	9.3 ± 0.1	8.8 ± 0.1 *	48.0 ± 0.4	49.6 ± 0.3 *	8.1 ± 0.3	9.1 ± 0.1	49.1 ± 0.6	48.9 ± 0.2
Sex (% male)	51.2 ± 1.4	49.2 ± 1.4	56.6 ± 1.0	45.7 ± 0.7 *	43.9 ± 3.0	50.9 ± 0.9 *	37.5 ± 1.9	52.3 ± 0.4 *
Ethnicity (% White)	69.5 ± 1.7	66.7 ± 1.8	79.2 ± 1.1	71.7 ± 1.3 *	64.7 ± 3.5	68.5 ± 1.4	68.3 ± 2.2	75.9 ± 1.0 *
Education (% university grad)	40.2 ± 1.6	48.7 ± 1.6 *	33.4 ± 1.4	42.0 ± 1.2 *	58.6 ± 3.1	42.9 ± 1.3 *	50.1 ± 2.3	36.5 ± 1.0 *
Marital status (% married or co-habiting)	N/A	N/A	61.7 ± 1.2	64.3 ± 1.1	N/A	N/A	68.3 ± 2.0	62.3 ± 0.9 *
Food secure (% yes)	84.0 ± 1.2	83.2 ± 1.2	87.3 ± 0.8	89.4 ± 0.7 *	87.4 ± 2.4	83.1 ± 0.9	91.8 ± 1.1	87.9 ± 0.6 *
BMI (kg/m^2^)	N/A	N/A	27.5 ± 0.2	27.2 ± 0.2	N/A	N/A	26.5 ± 0.3	27.5 ± 0.1
BMI z-score ^1^	0.4 ± 0.1	0.5 ± 0.2	N/A	N/A	0.3 ± 0.1	0.5 ± 0.1	N/A	N/A
Overweight/obese (% yes)	27.1 ± 1.6	26.3 ± 1.3	63.8 ± 1.5	60.3 ± 1.4	24.7 ± 2.9	26.9 ± 1.1	56.7 ± 2.8	62.6 ± 1.1 *
Urban residence (% yes)	81.3 ± 1.4	82 ± 1.3	80.5 ± 1.0	84.0 ± 1.0 *	78.1 ± 3.1	82.1 ± 1.1	86.3 ± 1.4	82.0 ± 0.8
Immigrant to Canada (% yes)	7.4 ± 0.8	9.2 ± 0.9	24.3 ± 1.3	29.4 ± 1.2 *	11.0 ± 2.0	8.0 ± 0.6	34.6 ± 2.2	26.0 ± 1.0 *

Values are presented as mean ± SE or % ± SE. * Significance differences between consumers and non-consumers at 5% level of significance; ^1^ BMI z-score was calculated for children 5–18 years.

**Table 3:** Percent energy and nutrient contribution of red and processed meat and plant-based meat alternatives to the daily nutrient intake of the red and processed meat consumers and the plant-based alternatives consumers.

Nutrients	Red and Processed Meat Consumers (*n* = 14,174,591)			Plant-Based Meat Alternatives Consumers (*n* = 4,926,277)		
	Total Daily Intake	% Contribution from Red and Processed Meat	95% CI	Total Daily Intake	% Contribution from Plant-Based Meat Alternatives	95% CI
**Energy (kcal)**	1927.2 ± 18.4	12.0 ± 0.2	(11.5, 12.4)	1776.2 ± 27.2	12.7 ± 0.6	(11.5, 13.8)
**Macronutrients**						
**Protein (g)**	80.9 ± 0.8	29.0 ± 0.4 ^4^	(28.1, 29.8)	71.7 ± 1.7	14.9 ± 0.6	(13.8, 16.0)
**Total Fat (g)**	70.0 ± 0.9	18.6 ± 0.3	(18.0, 19.3)	67.6 ± 1.3	21.4 ± 0.9 ^3^	(19.6, 23.1)
SFA (g)	24.5 ± 0.3	21.2 ± 0.4	(20.4, 22.0)	20.1 ± 0.4	13.0 ± 0.6	(11.8, 14.2)
MUFA (g)	25.8 ± 0.3	22.4 ± 0.4 ^5^	(21.7, 23.1)	25.0 ± 0.6	24.8 ± 1.0 ^2^	(23.0, 26.7)
PUFA (g)	13.4 ± 0.2	9.9 ± 0.2	(9.4, 10.3)	16.5 ± 0.5	27.0 ± 1.1 ^1^	(24.9, 29.1)
Cholesterol (mg)	284.8 ± 5.3	32.6 ± 0.5 ^3^	(31.6, 33.6)	205.1 ± 8.1	0.37 ± 0.09	(0.19, 0.54)
**Carbohydrates (g)**	232.9 ± 2.3	0.86 ± 0.03	(0.79, 0.92)	221.4 ± 3.9	7.2 ± 0.4	(6.4, 8.0)
Total Sugar (g)	93.9 ± 1.3	0.48 ± 0.02	(0.43, 0.52)	87.2 ± 1.8	4.40 ± 0.29	(3.82, 4.97)
Dietary Fibre (g)	14.7 ± 0.2	0.07 ± 0.02	(0.04, 0.11)	21.5 ± 0.4	20.1 ± 0.9 ^5^	(18.4, 21.8)
**Micronutrients: Vitamins**						
**Vitamin B12 (mcg)**	4.6 ± 0.1	38.0 ± 0.6 ^1^	(36.8, 39.2)	2.9 ± 0.1	1.6 ± 0.3	(1.1, 2.1)
**Vitamin D (mcg)**	4.8 ± 0.1	9.7 ± 0.4	(9.0, 10.4)	4.7 ± 0.2	0.0 ± 0.0	(0.0, 0.0)
**DFE (mcg)**	447.6 ± 5.6	1.76 ± 0.06	(1.64, 1.88)	445.0 ± 9.6	14.8 ± 0.9	(13.0, 16.6)
**Vitamin C (mg)**	98.4 ± 2.4	1.06 ± 0.11	(0.85, 1.27)	107.7 ± 3.8	2.62 ± 0.66	(1.32, 3.93)
**Vitamin A (mcg)**	619.7 ± 14.5	1.02 ± 0.10	(0.84, 1.21)	665.2 ± 22.5	0.68 ± 0.14	(0.41, 0.95)
**Micronutrients: Minerals**						
**Zinc (mg)**	11.2 ± 0.1	33.1 ± 0.5 ^2^	(32.1, 34.1)	8.9 ± 0.2	17.7 ± 0.7	(16.2, 19.1)
**Iron (mg)**	12.6 ± 0.1	15.0 ± 0.3	(14.4, 15.5)	12.2 ± 0.2	16.2 ± 0.7	(14.8, 17.7)
**Sodium (mg)**	2952.3 ± 34.5	13.3 ± 0.3	(12.7, 13.9)	2252.0 ± 46.6	6.8 ± 0.6	(5.5, 8.0)
**Potassium (mg)**	2601.7 ± 25.8	13.2 ± 0.2	(12.7, 13.6)	2727.7 ± 49.7	12.0 ± 0.7	(10.7, 13.3)
**Magnesium (mg)**	271.7 ± 2.6	8.5 ± 0.2	(8.2, 8.9)	351.5 ± 6.9	20.3 ± 0.8 ^4^	(18.8, 21.8)
**Calcium (mg)**	830.8 ± 12.0	2.69 ± 0.07	(2.55, 2.83)	832.2 ± 17.7	8.4 ± 0.5	(7.5, 9.4)

Values are presented as mean ± SE. #The numbers (1 to 5) represent the top five nutrients provided by red and processed meat and plant-based meat alternatives. SFA: saturated fatty acids; MUFA: monounsaturated fatty acids; PUFA: polyunsaturated fatty acids. DFE: dietary folate equivalents.

**Table 4:** Comparison of daily energy and nutrient intakes between the Canadian Community Health Survey (CCHS) 2015 and the simulated results among Canadians aged 1 year and older.

	Nutrients	Actual Intake (*n* = 34,005,286)	Simulated Intake ^#^ (*n* = 34,005,286)	Difference
		Mean ± SE	Mean ± SE	
Unchanged	Energy (kcal)	1863.4 ± 12.0	1867.1 ± 12.6	+3.7
	Carbohydrates (g)	226.3 ±1.4	230.2 ± 1.5	+3.9
	Total Sugar (g)	90.9 ± 0.8	91.8 ± 0.8	+0.9
	Total Fat (g)	68.8 ± 0.6	70.2 ± 0.7	+1.4
	SFA (g)	22.8 ± 0.2	22.1 ± 0.2	−0.7
	MUFA (g)	25.5 ± 0.3	26.2 ± 0.3	+0.7
	Potassium (mg)	2627.0 ± 16.9	2625.0 ± 17.9	−2.0
	Iron (mg)	12.3 ± 0.1	12.3 ± 0.1	0
	Sodium (mg)	2691.3 ± 22.3	2611.3 ± 21.3	−80
	Calcium (mg)	816.4 ± 7.5	830.4 ± 7.7	+14
	Vitamin C (mg)	101.2 ± 1.5	101.2 ± 1.2	0
	Vitamin A (mcg)	642.6 ± 9.7	634.3 ± 8.3	−8.3
	Vitamin D (mcg)	4.90 ± 0.07	4.82 ± 0.07	−0.08
Significant decrease *	Protein (g)	77.8 ± 0.6	73.4 ± 0.6	−4.4
	Cholesterol (mg)	261.8 ± 3.8	239.0 ± 3.8	−22.8
	Zinc (mg)	10.3 ± 0.1	9.4 ± 0.1	−0.9
	Vitamin B12 (mcg)	4.05 ± 0.06	3.46 ± 0.05	−0.59
Significant increase *	Dietary Fibre (g)	16.8 ± 0.2	18.1 ± 0.2	+1.3
	PUFA (g)	14.3 ± 0.2	15.6 ± 0.2	+1.3
	Magnesium (mg)	297.6 ± 2.1	315.4 ± 2.7	+17.8
	DFE (mcg)	437.5 ± 3.5	454.8 ± 3.8	+17.3
	NRF	517.4 ± 1.8	525.8 ± 1.8	+8.4

Values are presented as mean ± SE. ^#^ The simulated intake was derived by doubling the intake of plant-based meat alternatives and reducing the intake of red meat by half in CCHS 2015. * Significance differences between the CCHS 2015 and simulated data at a 5% level of significance. SFA: saturated fatty acids; MUFA: monounsaturated fatty acids; PUFA: polyunsaturated fatty acids. DFE: dietary folate equivalents; NRF: Nutrient Rich Food.

## Abbreviations

PUFA	polyunsaturated fatty acids
MUFA	monounsaturated fatty acids
SFA	saturated fatty acids
CCHS	Canadian Community Health Survey
BMI	body mass index
CAPI	computer-assisted personal interview
CATI	computer-assisted telephone interview
NRF	Nutrient Rich Index
